# Nutritional characterization and postprandial glycemic response of low-calorie date snacks incorporating psyllium and oat fibers in healthy and type 2 diabetes

**DOI:** 10.3389/fnut.2026.1862658

**Published:** 2026-06-16

**Authors:** Abdulmalik Alhuwaymil, Hani A. Alfheeaid, Metab Algeffari, Hassan Barakat

**Affiliations:** 1 Department of Food Science and Human Nutrition, College of Agriculture and Food, Qassim University, Buraydah, Saudi Arabia; 2 Department of Family and Community Medicine, College of Medicine, Qassim University, Buraydah, Saudi Arabia; 3 Abdullah Al-Othaim Diabetes Center, Medical City, Qassim University, Buraydah, Saudi Arabia

**Keywords:** food supply, functional foods, low-calorie date snacks, postprandial glycemic response, type 2 diabetes

## Abstract

**Background:**

The global rise in type 2 diabetes (T2D) and obesity, driven by high-sugar snacks, underscores the need for low-calorie, fiber-enriched alternatives that attenuate postprandial glycemia while maintaining cultural appeal in date-consuming regions.

**Objectives:**

This study nutritionally characterized low-calorie date-based snacks (LCDBSs) formulated with Sagai/Sukkari date paste (1:1), nuts, and oat or psyllium fibers at 5%, 7.5%, or 10% (OF1-3, PF1-3).

**Methods:**

The formulated snacks were organolyptically and nutritionally evaluated as well as postprandial glycemic responses in 20 healthy adults (fasting glucose < 100 mg/dL, BMI average 23.39 kg m^−^^2^) and 20 T2D patients (HbA1c 6.5−9.0%, fasting glucose 126-171 mg/dL) via standardized 100 g portions of sensorily preferred 7.5% variants (OF2, PF2) versus control (CF), measuring capillary glucose at 0, 30, 60, 90, and 120 min was examined.

**Results:**

Proximate composition revealed dose-dependent increases in dietary fiber (12.61–17.55% vs. 7.86% in CF), reductions in available carbohydrates (48.24–52.06% vs. 55.16% in CF), and energy (349.72–368.31 kcal 100 g^−1^ vs. 384.98 kcal 100 g^−1^ in CF). Bar incorporated psyllium boosting minerals like K (1203.89 mg 100 g^−1^ in PF3), Mg (181.06 mg 100 g^−1^ in PF1), and Fe (3.94 mg 100 g in PF3). Regarding sugar profile, fructose (4.78–6.25% vs. 8.27% in CF), and glucose (4.68–6.09% vs. 8.20% in CF). Across formulations, the snacks retained high phenolic contents and antioxidant activity (TPC 475–598 mg GAE 100 g^−1^; DPPH-RSA 616–773 μmol TE 100 g^−1^), exhibited nutritionally balanced amino-acid profiles (essential amino acids 383–441 and non?essential amino acids 509–566 mg 100 g^−1^; EAA: NEAA ratio 0.68–0.87), and maintained a favorable fatty-acid pattern dominated by oleic and linoleic acids (MUFA 36.82–37.7%, PUFA 23.89–26.36%, with SFA 36.24–39.09%). Both OF2 and PF2 significantly blunted glucose excursions compared with control (p < 0.05), with PF2 superior (lower peaks at 60–120 min in both groups), confirming a low glycemic potential.

**Conclusion:**

These findings position psyllium/oat-enriched LCDBSs as viable, culturally relevant options for glycemic control, weight management, and T2D dietary interventions.

## Introduction

The global prevalence of overweight, obesity, and type 2 diabetes (T2D) has risen sharply over recent decades, driven by rapid urbanization, sedentary lifestyles, and energy-dense dietary patterns (1). Excessive consumption of added sugars and refined carbohydrates, particularly from ultra-processed snacks and sugar-sweetened beverages (SSBs), is strongly associated with weight gain, insulin resistance, and an increased risk of T2D and cardiometabolic disease (2, 3). Snacking patterns have shifted toward frequent consumption of high-calorie, low-fiber cookies, candies, salty snacks, and soft drinks, further promoting positive energy balance and undermining glycemic control (4). In response, public health strategies increasingly emphasize replacing such products with convenient, nutrient-dense, high-fiber snacks that can attenuate postprandial glycemic excursions (5). Recent work on fruit-based snack bars and fiber-enriched baked goods demonstrates that reformulation with whole grains and functional fibers can lower glycemic index (GI) and enhance satiety, while remaining acceptable to consumers (6–8). Developing culturally appropriate, affordable snacks that meet these criteria is particularly relevant in regions where traditional carbohydrate-rich foods are central to the diet and T2D prevalence is high (8, 9).

Psyllium husk is a principally soluble, gel-forming fiber that markedly improves intestinal chyme viscosity, thereby slowing nutrient digestion and glucose absorption (10, 11). Meta-analyses and randomized controlled trials in individuals with metabolic syndrome and T2D show that psyllium supplementation improves fasting glucose and glycated hemoglobin, lowers serum LDL-cholesterol, and may contribute to modest reductions in body weight and waist circumference (10). Incorporating psyllium into solid foods, including cookies, has similarly been reported to decrease postprandial glycemic responses without adverse effects on micronutrient status, supporting its use as a functional ingredient in snack reformulation (12, 13). Similarly, oats are a rich source of β-glucan, a viscous, soluble fiber with well-established effects on postprandial glycemia, insulinemia, and appetite regulation (14). Acute feeding studies in healthy adults indicate that breakfasts or beverages containing oat β-glucan reduce early postprandial glucose and insulin peaks and can enhance subjective satiety, with effects that are dose- and viscosity-dependent (15). Regulatory assessments have recognized that sufficient intakes of oat β-glucan per unit of available carbohydrate can produce clinically relevant reductions in glycemic response, which has encouraged its incorporation into functional snack products such as cookies and bars (14, 16).

Date fruits (*Phoenix dactylifera* L.) are widely consumed in the Middle East and globally. They are increasingly used as natural sweeteners and binders in snack bars due to their high carbohydrate content, dietary fiber, minerals, and polyphenols (17, 18). Despite containing more than 70% sugars, many date varieties exhibit low to medium GI values, and clinical trials in individuals with prediabetes and T2D have shown that moderate daily date consumption does not adversely affect glycated hemoglobin or body weight and may improve lipid profiles (19). Recent reviews highlight the potential of date-based snack bars as functional foods for metabolic health, but also note that evidence on their glycemic impact, particularly when combined with added functional fibers, remains limited (20, 21).

Several studies have investigated the GI and satiety effects of fiber-rich cookies and fruit-based bars enriched with whole grains or soluble fibers, predominantly in healthy populations (9, 22). However, there is a paucity of data on low-calorie date-based snacks (LCDBSs) specifically formulated with psyllium and oat fibers, and, to our knowledge, no studies have systematically compared their nutritional profiles and glycemic indices in both healthy individuals and those with T2D under standardized testing conditions. This gap limits clinicians‘ and nutrition practitioners' ability to recommend culturally familiar products as evidence-based options for glycemic management. Therefore, this study aimed to characterize the nutritional properties of newly developed LCDBSs containing psyllium or oat fibers. In addition, the postprandial glycemic responses of these products were evaluated in healthy adults and individuals with T2D. By addressing these questions, the present work intends to provide data to support the development of culturally appropriate, fiber-enriched date snacks as feasible low-glycemic alternatives for both general and diabetic populations.

## Materials and methods

### Ingredients

All ingredients, such as Sagai date paste and Sukkari date paste, were purchased from Al Emtyaz Dates Factory, Buraydah, Qassim Region, SA (https://www.alemtyaz.com.sa) (accessed on May 5, 2024). At the same time, skimmed milk powder, whole oats, peanut butter, whole-wheat rusk, roasted sesame seeds, almonds, pecan nuts, dried coconut powder, edible salt, pure cow ghee, and rice oil were obtained from Al-Tamimi markets in Buraydah, Qassim Region, SA. At the same time, oat and psyllium fibers were purchased from the iHerb website (NuNaturals Oat Fiber Powder, 1 lb, 454 g, and Himalaya Organic Psyllium Husk Powder, 24 oz, 680 g), respectively. One serving of oat fiber (3 g) contains 0 g total fat, 0 mg cholesterol, 0 mg sodium, and 3 g dietary fiber, mainly insoluble, whereas one serving of psyllium fiber (6 g) contains 5 g dietary fiber, including 4 g soluble fiber and 1 g insoluble fiber (4:1).

### Preparation of LCDBSs

Following the supposed formulas in Table 1, six LCDBSs were formulated (three with oat fiber OF1: 5.0, OF2: 7.5, and OF3: 10%, and three with psyllium fiber PF1: 5.0, PF2: 7.5, and PF3: 10%), in addition to non-added-fiber formulas (CF). All dry ingredients except coconut shreds are mixed and roasted for 4–6 min with continuous stirring in a convection oven at 200 °C to prepare mixture 1. Coconut shreds are then added and stirred with the hot mixture for 2 min at the same temperature. Wet and liquid ingredients such as ghee, date paste, and peanut butter are mixed and stirred over heat until uniform to prepare mixture 2. Mixture 1 is combined with mixture 2 and thoroughly mixed in a mixer at speed 2 until homogeneous, then allowed to cool slightly and shaped. The shaped snacks (35 each) were then wrapped in polyethylene sheets and stored at 4 ± 1 °C until further analysis.

**Table 1:** Formulas of LCDBSs (g 100 g^−1^).

Ingredients	CF	LCDBSs g 100 g^−1^		
		OF1/PF1	OF2/PF2	OF3/PF3
Sukkari date paste Khalsa: Sukkari, (1:1)	55.00	50.00	50.00	50.00
Whole oat powder	10.0	10.0	7.50	5.0
Oat/psyllium fibers	0.00	5.00	7.50	10.0
Backed shabora powder	7.75	7.75	7.75	7.75
Shimmed milk powder	5.00	5.00	5.00	5.00
Cow's ghee: rice oil (1:1)	5.00	5.00	5.00	5.00
Coconut powder	4.50	4.50	4.50	4.50
Peanut butter	4.00	4.00	4.00	4.00
Pecan nut	3.00	3.00	3.00	3.00
Sesam seeds	3.00	3.00	3.00	3.00
Almond nut	2.50	2.50	2.50	2.50
Edible salt	0.25	0.25	0.25	0.25

CF, Control formula; OF1, Formula contain 5.0% oat fiber; OF2, Formula contain 7.5% oat fiber; OF3, Formula contain 10.0% oat fiber; PF1, Formula contain 5.0% psyllium fiber; PF2, Formula contain 7.5% psyllium fiber; and PF3, Formula contain 10.0% psyllium fiber.

### Organoleptical analysis of different LCDBSs

A 9-point hedonic scale was applied to the prepared snacks to identify the most acceptable formulations among trained panelists consisting of faculty members and postgraduate students, following the method described by Showkat et al. (23). The snacks were evaluated based on appearance, color, taste, aroma, texture, ease of cutting, and overall acceptability. Responses were recorded on a scale from “9 = extremely like” to “1 = extremely dislike.” Complete chemical analyses were conducted on all manufactured snack prototypes. However, clinical tests assessing glycemic indices were performed only on the most preferred samples selected through sensory evaluation, specifically those containing 7.5% oat fiber or psyllium, for the second phase of the study.

### Dietary composition and trace element content of different LCDBSs

The nutritional composition of all LCDBSs was thoroughly analyzed using standard AOAC methods. Moisture content was determined following AOAC 925.10, crude protein via AOAC 981.10, crude fat according to AOAC 963.15, ash per AOAC 942.05, total dietary fiber by AOAC 992.16, available carbohydrates calculated by difference, and energy value derived from proximate data (24). Sodium and potassium levels were quantified by flame photometry (Cambridge, UK) as per AOAC 970.35. Concentrations of calcium, magnesium, iron, copper, manganese, and zinc were measured using atomic absorption spectroscopy on a PerkinElmer Analyst™ 400 (Waltham, MA, USA), following AOAC 984.27 with element-specific hollow-cathode lamps and calibration curves (24). Phosphorus was assessed colorimetrically using the method of Borah et al. (25) with absorbance read at 880 nm on a Shimadzu UV-1,800 spectrophotometer (Kyoto, Japan). All analyses were conducted in triplicate, incorporating calibration standards and quality control samples in each run.

### Sugar profile of different LCDBSs

Sugar profiles in LCDBSs were determined by high-performance liquid chromatography (HPLC) using a Smartline system (Knauer, Germany) coupled with a refractive index detector (RID). The analysis followed established, validated protocols described in the published literature. Each sample consisted of 2 g, precisely weighed, dispersed in 10 mL of water. After vortex mixing for 5 min and sonication for 15 min to fully extract sugars, the suspension was centrifuged at 5,000 rpm for 10 min. The clear supernatant was passed through a 0.45 μm syringe filter prior to analysis. Separation was performed on a HILIC NH_2_ column (250 × 4.6 mm, 5 μm) maintained at 35 °C, injecting 20 μL aliquots. The isocratic mobile phase was 80:20 (v/v) acetonitrile: HPLC-grade water, delivered at 1.3 mL min^−1^. The RID operated at 20 °C and 29% relative humidity, identifying and quantifying fructose, glucose, sucrose, and maltose by peak area. Standards of each sugar at known concentrations generated calibration curves with strong linearity (R^2^ > 0.989) across the working range (26).

### Bioactive compounds analysis of different LCDBSs

Total phenolic content (TPC) in the LCDBSs was measured using the Folin-Ciocalteu assay and reported as mg gallic acid equivalents (GAE) per 100 g dry weight, following the procedure outlined by Bettaieb et al. (27). Antioxidant activity was assessed using the DPPH radical scavenging assay (DPPH-RSA), a standard colorimetric method based on 2,2-diphenyl-1-picrylhydrazyl (DPPH) free radicals. Inhibition percentage was calculated relative to a Trolox calibration curve, expressing results as μmol Trolox equivalents (TE) per g dry weight (μmol TE g^−1^) (28). Total carotenoids (TC) were determined colorimetrically with a modified protocol (29). Total flavonoids (TF) were quantified using protocols from Barakat & Almundarij (30), and expressed as mg quercetin equivalents (QE) per g dry weight (mg QE g^−1^).

### Polyphenols quantification in different LCDBSs

Phenolic compounds in various low-calorie date-based snacks (LCDBSs) were profiled following the method of Schneider (31), using an Agilent 1,260 Infinity HPLC (Agilent Technologies, Palo Alto, CA, USA). The setup included a quaternary pump, autosampler, and variable wavelength detector (VWD; Hewlett Packard 1,050) monitoring at 280 nm. Separation was achieved on a Kinetex^®^ EVO C18 column (1.7 μm, 150 × 4.6 mm; Phenomenex, Torrance, CA, USA) maintained at 30 °C. Elution followed a ternary linear gradient using mobile phases A, 0.1% trifluoroacetic acid (TFA) in HPLC water; B, acetonitrile; and C, HPLC methanol, beginning at 94:3:3 (v/v) for phenolics or 98:1:1 (v/v) for flavonoids. Samples (20 μL) were injected automatically at a flow rate of 1 mL min^−1^, with the column at 30 °C and the lab ambient at 20 °C. Individual phenolic peaks were quantified as mg kg^−1^ against external standard curves prepared from authentic compounds.

### Amino acid profiling in different LCDBSs

Amino acid (AA) profiles of LCDBSs were determined with a Sykam amino acid analyzer (Sykam GmbH, Eresing, Bavaria, Germany). This setup featured a S 2,100 quaternary pump for solvent delivery, S 5,200 autosampler, S 4,300 reaction module with dual-filter photometer (440–570 nm detection with integrated signal processing), and S 4,130 refrigerated reagent organizer. Separation was performed using an LCA K06/Na column under gradient conditions. Buffer A comprised 11.8 g trisodium citrate dihydrate, 6 g citric acid, 65 mL methanol, 6.5 ml 32% HCl, and 0.5 g phenol per liter (pH 3.45 N; 0.12 N). Buffer B included 19.6 g trisodium citrate dihydrate, 3.1 g NaOH, and 5 g boric acid per liter (pH 10.85 N; 0.20 N). Analyses were run at 1 ml/min with the column temperature ramped from 57 to 74 °C, and peaks were monitored at 440/570 nm. The standard mix (Sykam Type H, CAT No. S000029) contained 18 AAs at 2.5 μmol ml^−1^, with cystine at 1.25 μmol ml^−1^, in 0.1 N HCl containing 0.1% phenol; working standards were prepared by diluting 60 μL into 1.5 ml HPLC water, filtering through a 0.22 μm filter, and injecting 100 μL. For samples, ~1 g (to four decimals) underwent acid hydrolysis (20 ml 6 N HCl, 110 °C, 16 h in sealed tubes), followed by filtration, evaporation to dryness, resuspension in 100 mL HPLC water, 1:10 dilution, 0.22 μm filtration, and 100 μL injection. Quantification relied on relative response factors from standards, as described by Cohen et al. (32). Biological value and AA scores were calculated according to the WHO (2007) criteria and the approach of Chavan et al., using the standard equations.

### Fatty acid patterns and composition in different LCDBSs

Following the protocol of Aldai et al. (33), total fatty acids (FAs) from the LCDBSs samples were derivatized to their methyl esters (FAMEs). These FAMEs underwent analysis by gas-liquid chromatography (GLC) with flame ionization detection (FID). Separations were performed using a temperature program with an initial hold at 100 °C, followed by a ramp to 200 °C at 2 °C/min, then a final 10 min isothermal hold. Injector temperature was set to 250 °C, and the detector to 300 °C. Peak data and integration were handled through Saturn GC Workstation Software v5.51.

### Experimental design of clinical trial on selected LCDBSs

All experimental protocols were approved by the Institutional Review Board at Qassim University (Reference No. 25-10-18, issued Thursday, October 30, 2025). All participants provided written informed consent, and participation was voluntary, in full compliance with institutional and national guidelines.

### Healthy participants

Healthy adults aged 18–50 years were recruited from Qassim University campus and the local community via posters and email announcements. Eligible participants had fasting capillary blood glucose < 100 mg dL^−1^ (measured by finger-prick Accu-Chek glucometer), BMI average 23.39 kg m^−^^2^ [calculated as weight (kg)/height (m)^2^ under standardized conditions], and no history of diabetes, gastrointestinal disorders, or allergies to dates/ingredients. Exclusion criteria included pregnancy/lactation, smoking, regular medication/supplements affecting glucose metabolism, and inability to fast for ≥ 10 h. After screening 26 volunteers, 20 eligible participants attended multiple laboratory sessions separated by a ≥ 1-day washout period. Each session started at 8:00 AM following overnight fasting (≥ 10 h), with standardized evening meals and no vigorous activity the prior day. Participants consumed either 100 g of a normal date bar or LCDBSs containing 7.5% fiber, either oat or psyllium (OF2/PF2). Capillary blood glucose was measured at baseline (0 min) and 30, 60, 90, and 120 min post-ingestion.

### Type 2 diabetes participants

Adults aged 18–65 years diagnosed with type 2 diabetes (T2D) were recruited from Abdullah Al-Othaim Diabetes Center, Medical City, Qassim University, via flyers, physician referrals, and social media announcements. Eligible participants had confirmed T2D (physician-diagnosed, HbA1c 6.5%−9.0%), fasting capillary blood glucose 126–171 mg dL^−1^ (measured by finger-prick Accu-Chek glucometer), BMI average 29.24 kg m^−2^, and stable glycemic control (no changes in antidiabetic medications for ≥ 3 months). Exclusion criteria included type 1 diabetes, pregnancy/lactation, insulin use, history of severe hypoglycemia/hyperosmolar states, gastrointestinal disorders, allergies to dates/ingredients, uncontrolled hypertension (BP >160/100 mmHg), or inability to fast for ≥ 8 h. After screening 28 volunteers, 20 eligible participants attended multiple laboratory sessions separated by a ≥1-day washout period. Each session began at 8:00 AM following an overnight fast of ≥8 h, with a standardized low-glycemic evening meal, no vigorous physical activity, and usual evening antidiabetic medications. Participants consumed either 100 g of a normal date bar or selected LCDBSs containing 7.5% fiber, either containing oat or psyllium fiber (OF2/PF2). Capillary blood glucose was measured at baseline (0 min) and 30, 60, 90, 120 min post-ingestion, with optional continuous glucose monitoring for safety.

### Statistical analysis

Statistical analysis. Data were analyzed using SPSS v22.0 (IBM Corp., Armonk, NY, USA) under a completely randomized design. Normality and homogeneity of variance were checked before analysis. One-way ANOVA was used to compare formulation means regarding organolyptical characteristics, proximate chemical composition, minerals content, and phytochemical contents, followed by Tukey's *post hoc* test at p < 0.05. For the sugar profile, polyphenolic compounds profiling, amino acids, fatty Acids profiling, a two-way ANOVA was used to test the effects of fiber type, concentration, and their interactions, as well as clinical glucose-response data was statistically studied for fiber type, time, and their interactions, with repeated-measures and simple main-effects comparisons applied where appropriate. Results are presented as mean ± SE following Steele et al. (34).

## Results and discussion

### Organolyptical characteristics of formulated LCDBSs

Organoleptic characteristics were assessed using a 9-point hedonic scale (*n* = 15 panelists) for appearance, odor, taste, texture, mouthfeel, bitterness, and overall acceptability, as illustrated in Table 2. The CF scored highest across attributes (6.73–6.93), indicating strong baseline acceptance. Comparatively, oat fiber-enriched bars showed lower scores than CF, particularly appearance (5.73–5.80 vs. 6.73, *p* < 0.05). OF2 excelled in taste (6.53, comparable to CF) and overall acceptability (6.27), but OF3 declined sharply in texture and mouthfeel, exhibiting 4.93 and 4.80, respectively. The psyllium-enriched bars averaged lower than CF, with appearance ~5.87–6.00 (*p* < 0.05). PF1 was rated highest among the enriched, while PF3 had the lowest texture (4.67) and overall acceptability; hence, no attribute matched CF significantly. Fiber levels increased, but the trend was downward for texture, mouthfeel, and overall scores. OF2 and PF2 were preferred, suggesting that 5%−7.5% of fiber could be optimal. The CF remained superior, and no significant (*p* > 0.05) differences in odor and appearance were observed.

**Table 2:** Organolyptical characteristics of different formulated LCDBSs containing psyllium or oat Fibers (mean ± SE), *n* = 15.

LCDBSs formulas	Organoleptical characteristics						
	Appearance	Odor	Taste	Texture	Mouthfeel	Bitterness	Overall acceptability
CF	6.73	6.93	6.33	6.20	6.60	6.87	6.73
	± 0.12 ^a^	± 0.18^a^	± 0.29^a^	± 0.28^a^	± 0.25^a^	± 0.29^a^	± 0.15^a^
OF1	5.73	5.93	5.47	5.33	5.67	5.93	5.73
	± 0.12^b^	± 0.18^b^	± 0.22^b^	± 0.21^bcd^	± 0.21^bc^	± 0.25^b^	± 0.15^bc^
OF2	5.80	6.13	6.53	5.87	6.33	6.13	6.27
	± 0.14^b^	± 0.19^b^	± 0.27^a^	± 0.26^ab^	± 0.25^ab^	± 0.29^ab^	± 0.25^ab^
OF3	5.80	6.00	5.33	4.93	4.80	5.53	5.47
	± 0.14^b^	± 0.17^b^	± 0.25^b^	± 0.25^cd^	± 0.37^c^	± 0.31^b^	± 0.24^c^
PF1	5.87	5.93	5.87	5.67	5.73	5.87	5.87
	± 0.19^b^	± 0.23^b^	± 0.27^ab^	± 0.25^abc^	± 0.37^abc^	± 0.29^b^	± 0.26^bc^
PF2	5.87	5.73	5.33	5.40	5.07	5.33	5.67
	± 0.19^b^	± 0.18^b^	± 0.30^b^	± 0.27^bcd^	± 0.34^c^	± 0.36^b^	± 0.29^bc^
PF3	6.00	5.80	5.20	4.67	5.13	5.27	5.27
	± 0.17^b^	± 0.14^b^	± 0.28^b^	± 0.19^d^	± 0.29^c^	± 0.34^b^	± 0.27^c^

SE, Standard error; CF, Control formula; OF1, Formula contain 5.0% oat fiber; OF2, Formula contain 7.5% oat fiber; OF3, Formula contain 10.0% oat fiber; PF1, Formula contain 5.0% psyllium fiber; PF2, Formula contain 7.5% psyllium fiber; and PF3, Formula contain 10.0% psyllium fiber, ^a, b, c^, No significant difference (*p* > 0.05) between any two means within the same column with the same superscripted letters.

The organoleptic characteristics indicated acceptable viability despite nutritional alterations, but the OF2/PF2 balance provides health benefits with liking, supporting market potential for functional date snacks; therefore, they were used in the second phase of this study (8). The oat fiber bars exhibited lower appearance to beta-glucan dilution of date color/cohesion, consistent with oat-fortified snacks, which also cause a texture/mouthfeel drop as a signal of excessive hardness from hydration swelling patterns seen at high oat fiber levels (35, 36). Likewise, increasing fiber levels in psyllium bars led to declines in appearance, texture, mouthfeel, and overall acceptability, possibly resulting from greater firmness or grittiness (37, 38). Indeed, OF2/PF2 preferences exhibited a balance in nutrition (fiber↑, GI↓) with a liking >5 threshold for acceptability, as in KDBB2 (5–6 range selected) (8). Control formula superiority and acceptable enriched scores affirm viability despite alterations; OF2/PF2 health-liking synergy supports functional date-snacks, advancing to phase II per iterative trials.

### Proximate composition of formulated LCDBSs

The incorporation of oat and psyllium fibers significantly modified the proximate composition of LCDBSs (Table 3). Compared with the control formula (CF), all fiber-enriched treatments exhibited lower moisture and protein contents, markedly higher dietary fiber, and reduced available carbohydrates and energy values. In contrast, total fat and ash contents remained statistically unchanged. Moisture decreased from 11.31% in CF to 9.93%−10.47% in the fiber-fortified formulations, indicating a slight reduction in free water content after fiber incorporation. Protein showed a modest decline from 8.20% in CF to 7.18%−7.84% in the fortified snacks, most likely reflecting a dilution effect from replacing date paste and oat powder with non-protein fiber ingredients. The most pronounced change was observed in dietary fiber, which increased from 7.86% in CF to 12.61%−17.48% in oat-fortified snacks and to 13.10%−17.55% in psyllium-fortified snacks. This increase was clearly dose-dependent for both fiber sources, with the highest inclusion level (10%) producing the greatest enrichment. In parallel, available carbohydrates declined from 55.16% in CF to 48.24%−52.06% across fortified formulations, and this reduction was accompanied by a decrease in the calculated energy value from 384.98 to 349.72–368.31 kcal 100 g^−1^.

**Table 3:** Proximate chemical composition of different formulated LCDBSs containing psyllium or oat Fibers (mean ± SE), *n* = 3.

Composition^*^	LCDBSs formulas						
	CF	OF1	OF2	OF3	PF1	PF2	PF3
Moisture	11.31	10.47	10.19	10.08	10.18	10.19	9.93
	± 0.25^a^	± 0.32^b^	± 0.04^b^	± 0.12^b^	± 0.17^b^	± 0.19^b^	± 0.09^b^
Protein	8.20	7.84	7.40	7.18	7.63	7.41	7.23
	± 0.18^a^	± 0.04^ab^	± 0.16^bc^	± 0.18^c^	± 0.32^bc^	± 0.09^bc^	± 0.15^c^
Total fat	14.62	14.36	14.30	14.23	14.38	14.40	14.18
	± 0.27^a^	± 0.19^a^	± 0.06^a^	± 0.19^a^	± 0.09^a^	± 0.10^a^	± 0.10^a^
Ash	2.85	2.80	2.69	2.79	2.66	2.53	2.52
	± 0.06^a^	± 0.09^a^	± 0.05^a^	± 0.11^a^	± 0.11^a^	± 0.07^a^	± 0.16^a^
Dietary fiber	7.86	12.61	14.92	17.48	13.10	15.45	17.55
	± 0.35^d^	± 0.10^c^	± 0.10^b^	± 0.31^a^	± 0.08^c^	± 0.20^b^	± 0.23^a^
Available carbohydrates	55.16	51.92	50.50	48.24	52.06	50.03	48.60
	± 0.55^a^	± 0.45^b^	± 0.13^c^	± 0.09^d^	± 0.45^b^	± 0.31^c^	± 0.17^d^
Energy (kcal)	384.98	368.31	360.30	349.72	368.12	359.32	350.9
	± 5.34^a^	± 0.24^b^	± 0.56^c^	± 1.32^d^	± 0.54^b^	± 0.82^c^	± 0.37^d^

^*^Presented on 100 wet weight; SE, Standard error; CF, Control formula; OF1, Formula contain 5.0% oat fiber; OF2, Formula contain 7.5% oat fiber; OF3, Formula contain 10.0% oat fiber; PF1, Formula contain 5.0% psyllium fiber; PF2, Formula contain 7.5% psyllium fiber; and PF3, Formula contain 10.0% psyllium fiber; ^a, b, c^, No significant difference (*p* > 0.05) between any two means within the same row with the same superscripted letters.

These findings confirm that enriching snacks with either oat or psyllium fiber effectively reduced their caloric density while substantially increasing their fiber content, which may improve acute postprandial glucose tolerance and enhance glycemic regulation after a subsequent lunch in healthy young adults (14–16). At comparable inclusion levels, oat and psyllium formulations displayed closely similar proximate profiles, indicating that fiber level exerted a greater effect than fiber type on these parameters (39, 40). The preservation of total fat and ash contents suggests that the reformulation selectively altered carbohydrate-related fractions without disturbing overall lipid or mineral residue content (41). From a nutritional standpoint, high-fiber, low-carbohydrate snacks improve glycemic control by slowing glucose absorption, boost satiety for weight management, and support insulin sensitivity, aligning with diabetes-friendly product recommendations (11, 42, 43).

### Minerales content in formulated LCDBSs

The mineral profiles of the formulated LCDBSs varied according to the type and level of added fiber (Table 4). Oat fiber fortification generally reduced several minerals relative to CF, particularly Na, Ca, P, and Zn, whereas psyllium-containing formulations retained or improved the concentrations of several nutritionally relevant minerals. The Na declined from 434.54 mg 100 g^−1^ in CF to 387.14 mg 100 g^−1^ in OF3, while Ca decreased from 282.29 to approximately 256–259 mg 100 g^−1^ in most oat formulations and PF3. 'The P also showed significant (*p* < 0.05) reduction in oat treatments, reaching 394.34 mg 100 g^−1^ in OF3 compared with 493.86 mg 100 g^−1^ in CF. In contrast, psyllium fortification increased levels of several macro- and microelements. The Mg increased from 168.72 mg 100 g^−1^ in CF to 181.06 mg 100 g^−1^ in PF1, while potassium increased progressively to 1,203.89 mg 100 g^−1^ in PF3. Likewise, Mn, Cu, Fe, and Se were consistently higher in psyllium formulations than in both CF and oat-based formulations, with iron increasing from 3.40 mg 100 g^−1^ in CF to 3.94 mg 100 g^−1^ in PF3, and Se increasing from 52.62 μg 100 g-1 in CF to 57.98 μg 100 g^−1^ in PF1.

**Table 4:** Minerales content in different formulated LCDBSs (mean ± SE), *n* = 3.

Minerals^*^	LCDBSs formulas						
	CF	OF1	OF2	OF3	PF1	PF2	PF3
Na	434.54	395.04	391.09	387.14	430.19	421.59	399.03
	± 17.37^a^	± 11.06^ab^	± 12.93^ab^	± 19.80^b^	± 14.22^ab^	± 16.94^ab^	± 17.19^ab^
Ca	282.29	258.03	256.63	257.83	279.47	273.88	259.02
	± 5.11^a^	± 7.76^b^	± 1.74^b^	± 4.76^b^	± 3.07^a^	± 2.99^a^	± 6.78^b^
Mg	168.72	166.66	164.6	161.51	181.06	179.25	175.66
	± 3.46^bc^	± 1.43^c^	± 1.40^c^	± 4.35^c^	± 3.64^a^	± 1.62^a^	± 3.56^ab^
P	493.86	459.42	448.96	394.34	488.91	479.14	469.89
	± 8.20^a^	± 9.70^cd^	± 6.54^d^	± 5.75^e^	± 3.13^ab^	± 4.98^abc^	± 6.85^bcd^
K	1119.96	1094.45	1105.36	1116.28	1168.02	1191.86	1203.89
	± 21.33^bc^	± 15.95^c^	± 11.11^c^	± 13.27^bc^	± 12.03^ab^	± 18.37^a^	± 17.55^a^
Mn	2.27	2.33	2.14	1.93	2.57	2.54	2.49
	± 0.03^b^	± 0.03^b^	± 0.03^c^	± 0.03^d^	± 0.04^a^	± 0.04^a^	± 0.04^a^
Cu	0.85	0.87	0.86	0.85	0.96	0.95	0.93
	± 0.01^b^	± 0.01^b^	± 0.01^b^	± 0.01^b^	± 0.01^a^	± 0.01^a^	± 0.01^a^
Zn	3.96	3.60	2.60	2.47	3.92	3.84	2.61
	± 0.06^a^	± 0.05^b^	± 0.04^c^	± 0.03^c^	± 0.06^a^	± 0.06^a^	± 0.04^c^
Fe	3.40	3.58	3.62	3.74	3.82	3.90	3.94
	± 0.05^d^	± 0.05^c^	± 0.05^c^	± 0.06^bc^	± 0.06^ab^	± 0.06^ab^	± 0.06^a^
Se (μg 100 ^−1^)	52.62	52.71	51.07	48.85	57.98	57.41	56.26
	± 0.77^b^	± 0.77^b^	± 0.74^bc^	± 0.71^c^	± 0.85^a^	± 0.84^a^	± 0.82^a^

^*^Presented on 100 wet weight, SE: Standard error, CF; Control formula; OF1, Formula contain 5.0% oat fiber; OF2, Formula contain 7.5% oat fiber; OF3, Formula contain 10.0% oat fiber; PF1, Formula contain 5.0% psyllium fiber; PF2, Formula contain 7.5% psyllium fiber; and PF3, Formula contain 10.0% psyllium fiber; ^a, b, c^,, No significant difference (*p* > 0.05) between any two means within the same row with the same superscripted letters.

These data indicate that psyllium not only preserved mineral density better than oat fiber but also, in several cases, improved the micronutrient profile of the final product (44). The nutritional relevance of these findings is considerable, especially for K, Mg, Se, and Fe, which are important for cardiovascular, metabolic, and antioxidant functions (12, 37). The higher K-to-Na ratio in psyllium-fortified snacks may be particularly beneficial for cardiometabolic health (45, 46). Overall, psyllium-enriched LCDBSs appear superior to oat-enriched counterparts with respect to mineral quality (44).

### Phytochemical contents in different formulated LCDBSs

The phytochemical analysis showed that all formulations retained high levels of phenolic compounds and antioxidant activity despite fiber enrichment (Table 5). The TPC ranged from 475.49 to 598.36 mg GAE 100 g^−1^, TF from 349.10 to 437.76 mg QE 100 g^−1^, and TC from 200.81 to 251.43 mg 100 g^−1^. The CF consistently exhibited the highest values, whereas both oat and psyllium fortification produced gradual numerical decreases, especially at higher inclusion levels. However, most of these decreases were statistically non-significant (*p* > 0.05), indicating that the date matrix remained the dominant contributor to the overall phytochemical load. The DPPH radical scavenging activity showed a clearer reduction pattern than TPC, TF, or TC. Antioxidant activity decreased from 773.25 μmol TE 100 g^−1^ in CF to 650.00 and 616.05 μmol TE 100 g^−1^ in OF3 and PF3, respectively.

**Table 5:** Phytochemical contents in different formulated LCDBSs (mean ± SE), *n* = 3.

Phytochemicals ^*^	LCDBSs formulas						
	CF	OF1	OF2	OF3	PF1	PF2	PF3
TPC [mg GAE 100 g^−1^]	598.36	561.54	530.32	501.24	525.01	495.9	475.49
	± 40.67^a^	± 34.05^a^	± 37.10^a^	± 30.67^a^	± 36.73^a^	± 39.340^a^	± 35.40^a^
DPPH-RSA [μmol of TE 100 g^−1^]	773.25	727.19	686.27	650.00	679.40	641.28	616.05
	± 20.75^a^	± 29.46^ab^	± 31.90^abc^	± 35.12^bc^	± 31.58^abc^	± 34.40^bc^	± 37.83^c^
TF [mg QE 100 g^−1^]	437.76	411.85	388.74	368.24	384.85	363.32	349.10
	± 17.41^a^	± 22.11^ab^	± 23.27^ab^	± 24.53^ab^	± 23.04^ab^	± 24.34^ab^	± 26.02^b^
TC [mg 100 g^−1^]	251.43	236.93	223.44	212.06	221.21	208.6	200.81
	± 14.24^a^	± 18.45^a^	± 16.97^a^	± 19.63^a^	± 16.80^a^	5 ± 15.72^a^	± 18.32^a^

^*^Presented on 100 wet weight; SE, Standard error; CF, Control formula; OF1, Formula contain 5.0% oat fiber; OF2, Formula contain 7.5% oat fiber; OF3, Formula contain 10.0% oat fiber; PF1, Formula contain 5.0% psyllium fiber; PF2, Formula contain 7.5% psyllium fiber; and PF3, Formula contain 10.0% psyllium fiber, ^a, b, c^,No significant difference (p > 0.05) between any two means within the same row with the same superscripted letters.

This result suggests that although fiber enrichment diluted antioxidant compounds on a weight basis, the formulations still retained substantial free-radical scavenging capacity (47, 48). The persistence of relatively high phytochemical values across treatments supports the functional potential of date-based snacks as antioxidant-rich products. Dates retain high phenolics, flavonoids, and antioxidant activity post-processing. Date bar studies show TPC and radical-scavenging activity persistence, confirming their functional snack potential (47, 49). Fiber enrichment increases dietary fiber intake and improves nutritional quality while largely preserving phenolic content and antioxidant activity. Although texture may change, bioactive retention supports better functional value (50). Since dates are intrinsically rich in phenolic antioxidants, the moderate decline observed after reformulation may be considered acceptable given the gains in fiber enrichment and carbohydrate reduction (51, 52).

### Sugar profile in formulated LCDBSs

The sugar profiles of the formulated LCDBSs were dominated by fructose and glucose, with sucrose and maltose present in smaller amounts (Table 6). In CF, fructose and glucose contents were 8.27 and 8.20%, respectively, confirming the predominance of reducing sugars typical of date-based products. Fiber fortification significantly decreased both sugars across treatments, with psyllium exerting a stronger effect than oat fiber. Mean fructose and glucose values in oat-fortified formulations were 6.25 and 6.09%, respectively, compared with 4.78 and 4.68% in psyllium-containing formulations. Sucrose remained relatively stable across treatments, ranging from 1.50 to 3.00%, and did not show significant differences among treatments (*p* > 0.05). Maltose declined more distinctly, especially in psyllium formulations at 7.5 and 10%, where it was not detected.

**Table 6:** Sugar profile in formulated LCDBSs.

Sugars ^*^	Fiber type	Fiber %				Mean
		CF	5%	7.5%	10%	
Fructose	O	8.27^aA^	6.03^aB^	5.00^aB^	5.68^aB^	6.25^a^
	P		3.96^bB^	3.27^bB^	3.62^bB^	4.78^b^
Glucose	O	8.20^aA^	5.89^aB^	4.87^aB^	5.38^aB^	6.09^a^
	P		3.84^bB^	3.33^aB^	3.33^bB^	4.68^b^
Sucrose	O	2.75^aA^	2.75^aA^	2.50^aA^	3.00^aA^	2.75^a^
	P		2.25^aA^	1.50^aA^	1.50^bA^	2.00^a^
Maltose	O	1.30^aA^	0.86^aA^	0.43^aA^	0.87^aA^	0.87^a^
	P		0.86^aA^	0.00^aB^	0.00^bB^	0.54^a^

^*^Presented on 100 wet weight; CF, Control formula; ^a, b, c^No significant difference (*p* > 0.05) between any two means within the same column with the same superscripted letters; and ^A, B, C^No significant difference (*p* > 0.05) between any two means within the same row with the same superscripted letters.

These results indicate that fiber substitution effectively reduced the concentration of rapidly available sugars, especially in psyllium-enriched products (53). The reduction in fructose and glucose complements the decline in available carbohydrates and energy observed in the proximate analysis (11, 13). From a functional perspective, this compositional shift may improve the glycemic performance of the formulated snacks, particularly when combined with the viscosity-related physiological effects of psyllium and oat soluble fibers (54–56).

### Polyphenolic compounds profiling of formulated LCDBSs

HPLC profiling revealed a diverse phenolic and flavonoid composition in the LCDBSs, with gallic acid, chlorogenic acid, caffeic acid, ferulic acid, and naringenin identified as the dominant compounds (Table 7). Among phenolic acids, gallic acid was the most abundant, ranging from 49.50 mg 100 g^−1^ in CF to 107.95 mg 100 g^−1^ in PF3. Chlorogenic acid varied between 19.60 and 33.25 mg 100 g^−1^, while caffeic acid ranged from 5.12 to 13.56 mg 100 g^−1^ and ferulic acid from 5.68 to 9.13 mg 100 g^−1^. Among flavonoids, naringenin showed particularly high values, reaching 53.10 mg 100 g^−1^ in PF3, while catechin ranged from 7.62 to 12.48 mg 100 g^−1^.

**Table 7:** Polyphenolic compounds profiling of Formulated LCDBSs.

Polyphenolics (mg 100 g^−1^)	Fiber type	Fiber %				Mean
		CF	5%	7.5%	10%	
Phenolics						
Gallic acid	O	49.50^aA^	73.05^aA^	80.28^aA^	56.47^bA^	64.83^a^
	P		60.46^aA^	79.16^aA^	107.95^aA^	74.27^a^
Chlorogenic acid	O	19.89^aA^	29.34^aA^	19.60^aA^	28.22^aA^	24.26^a^
	P		22.68^aA^	26.18^aA^	33.25^aA^	25.50^a^
Methyl gallate	O	0.48^aA^	0.57^aA^	0.00^aA^	0.00^aA^	0.26^a^
	P		0.00^bA^	0.00^aA^	0.00^aA^	0.12^a^
Caffeic acid	O	5.12^aA^	7.28^aA^	8.93^aA^	6.17^bA^	6.88^a^
	P		7.08^aAB^	12.08^aAB^	13.56^aA^	9.46^a^
Syringic acid	O	0.91^aB^	1.99^aAB^	3.21^aA^	2.99^aA^	2.28^a^
	P		1.45^aA^	1.64^bA^	1.40^bA^	1.35^a^
Ellagic acid	O	0.90^aA^	0.00^aA^	0.00^aA^	0.00^bA^	0.23^a^
	P		0.00^aA^	0.92^aA^	3.13^aA^	1.24^a^
Coumaric acid	O	0.45^aB^	7.60^aA^	5.96^aA^	5.72^aA^	4.93^a^
	P		2.89^bA^	2.73^bA^	3.31^bA^	2.35^a^
Vanillin	O	0.45^aB^	1.08^aAB^	1.60^aAB^	1.68^aA^	1.20^a^
	P		0.38^aA^	0.42^bA^	0.61^bA^	0.47^a^
Ferulic acid	O	6.92^aA^	7.47^aA^	7.67^aA^	5.68^bA^	6.94^a^
	P		7.52^aA^	9.13^aA^	8.70^aA^	8.07^a^
Rosmarinic acid	O	0.89^aA^	0.75^aA^	0.00^bA^	0.00^aA^	0.41^a^
	P		0.00^aA^	2.95^aA^	0.95^aA^	1.20^a^
Cinnamic acid	O	0.54^aA^	1.09^aA^	0.36^aA^	0.81^aA^	0.70^a^
	P		0.42^bA^	0.51^aA^	0.25^aA^	0.43^a^
Flavonoids						
Catechin	O	10.64^aA^	7.91^aA^	7.62^bA^	8.59^bA^	8.69^a^
	P		9.39^aA^	10.28^aA^	12.48^aA^	10.70^a^
Rutin	O	0.00^aA^	0.00^aA^	0.00^aA^	0.00^bA^	0.00^a^
	P		0.00^aA^	0.00^aA^	2.11^aA^	0.53^a^
Naringenin	O	21.39^aA^	24.15^aA^	33.66^aA^	18.94^bA^	24.54^a^
	P		23.57^aA^	27.47^aA^	53.10^aA^	31.38^a^
Daidzein	O	0.89^aA^	0.61^aA^	0.82^aA^	0.90^bA^	0.81^aA^
	P		2.02^aA^	6.84^aA^	6.75^aA^	4.13^aA^
Quercetin	O	1.03^aA^	1.22^aA^	1.29^aA^	2.32^aA^	1.47^a^
	P		0.00^bA^	1.07^aA^	1.04^bA^	0.79^a^
Kaempferol	O	0.00^aA^	0.00^aA^	0.00^aA^	0.00^bA^	0.00^a^
	P		0.00^aA^	0.00^aA^	1.12^aA^	0.28^a^
Hesperidin	O	0.00	0.00	0.00	0.00	0.00
	P		0.00	0.00	0.00	0.00

^*^Presented on 100 wet weight; CF, Control formula; ^a, b, c^ No significant difference (*p* > 0.05) between any two means within the same column with the same superscripted letters; and ^A, B, C^No significant difference (*p* > 0.05) between any two means within the same row with the same superscripted letters.

Different fibers (psyllium, oat, inulin, pea) variably alter proximate composition and phenolics in fruit snacks, with carrot boosting TPC the most. Fiber type affects phenolic distribution more than total phenolic content, supporting tailored selection for target profiles (49, 57). Psyllium generally preserves or enhances several phenolic compounds better than oat fiber, especially gallic acid, caffeic acid, naringenin, and daidzein (49, 50). Oat fiber, on the other hand, showed stronger enrichment in coumaric acid and vanillin at certain inclusion levels (50). Several compounds, including rutin and kaempferol, were absent or present only in trace amounts. These results demonstrate that although total phenolics tended to decline with fiber enrichment, the detailed phenolic fingerprint remained rich and diverse (48, 58). The retention of compounds such as gallic, caffeic, and ferulic acids, together with naringenin and catechin, is particularly important because these molecules are strongly associated with antioxidant and anti-inflammatory activity (57). Thus, the formulated LCDBSs retain appreciable polyphenolic activity even after reformulation.

### Amino acids profiling of formulated LCDBSs

The amino acid composition of the LCDBSs indicated that all formulations contained both essential (EAAs) and non-essential amino acids (NEAAs) in nutritionally relevant proportions (Table 8). The EAAs ranged from 383.36 to 440.88 mg 100 g^−1^, while the NEAAs ranged from 509.10 to 565.74 mg 100 g^−1^. The EAAs/NEAAs ratio varied between 0.68 and 0.87, with the highest value observed in OF2. Leucine, lysine, threonine, and phenylalanine were the predominant essential amino acids, while glutamic acid, arginine, and aspartic acid were the major non-essential amino acids. Oat fortification at 7.5% yielded the highest EAA content (440.88 mg 100 g^−1^), whereas psyllium formulations maintained relatively stable EAA values around 400 mg 100 g^−1^. Histidine showed considerable variability across treatments, reaching 150.51 mg 100 g^−1^ in OF2, while lysine was highest in PF2 (54.42 mg 100 g^−1^).

**Table 8:** Amino acids (mg 100 g^−1^) profiling of Formulated LCDBSs.

Amino acids	Fiber type	Fiber %				Mean
		CF	5%	7.5%	10%	
Essential amino acids						
Lysine	O	46.80^aA^	45.02^aA^	41.32^aA^	43.93^bA^	44.27^a^
	P		40.47^aB^	46.96^aAB^	54.42^aA^	47.16^a^
Threonine	O	62.13^aA^	61.15^aA^	65.13^aA^	65.95^bA^	63.59^a^
	P		59.91^aB^	64.23^aB^	72.65^aA^	64.73^a^
Valine	O	35.07^aA^	27.58^aA^	28.21^aA^	43.69^aA^	33.64^a^
	P		30.77^aA^	36.56^aA^	30.45^bA^	33.21^a^
Methionine	O	14.77^aA^	11.61^aBC^	10.98^aC^	13.27^aAB^	12.66^a^
	P		10.62^aB^	12.43^aAB^	12.34^aAB^	12.54^a^
Isoleucine	O	29.11^aA^	27.71^aA^	26.08^aA^	24.12^aA^	26.76^a^
	P		22.79^bA^	24.60^aA^	26.26^aA^	25.69^a^
Leucine	O	55.70^aA^	58.46^aA^	62.36^aA^	63.16^aA^	59.92^a^
	P		63.17^aA^	67.37^aA^	54.70^aA^	60.24^a^
Phenylalanine	O	41.56^aA^	40.42^aA^	41.19^aA^	49.53^aA^	43.18^a^
	P		43.39^aA^	44.57^aA^	38.45^aA^	41.99^a^
Histidine	O	81.41^aA^	113.08^aA^	150.51^aA^	99.32^aA^	111.08^a^
	P		124.98^aA^	92.12^bA^	99.24^aA^	99.44^b^
Cysteine	O	16.81 ^aA^	^aA^15.68	^aA^15.10	^aA^14.72	15.58^a^
	P		^aA^15.22	^aA^13.26	^aA^12.72	14.50^a^
Non-essential amino acids						
Arginine	O	109.35^aA^	104.78^aA^	108.02^aA^	110.63^aA^	108.20^a^
	P		102.32^aA^	98.49^aA^	96.48^bA^	101.66^a^
Aspartic acid	O	86.24^aA^	100.88^aA^	83.36^aA^	74.24^bA^	86.18^a^
	P		92.74^aA^	94.49^aA^	97.07^aA^	92.64^a^
Serine	O	48.45^aA^	42.69^aA^	44.07^aA^	49.73^aA^	46.24^a^
	P		44.22^aA^	46.15^aA^	44.94^aA^	45.94^a^
Glutamic acid	O	172.25^aAB^	186.41^aA^	161.94^aBC^	152.21^aC^	168.20^a^
	P		176.05^aA^	169.60^aA^	155.14^aA^	168.26^a^
Proline	O	40.61^aA^	31.34^aAB^	32.27^aAB^	27.51^bB^	32.93^a^
	P		29.24^aB^	33.17^aAB^	36.81^aAB^	34.96^a^
Glycine	O	46.67^aA^	34.93^aA^	31.42^aA^	46.43^aA^	39.86^a^
	P		35.07^aA^	42.90^aA^	40.13^aA^	41.19^a^
Alanine	O	43.72^aA^	35.14^aB^	35.44^bB^	46.05^bA^	40.09^a^
	P		39.19^aB^	43.02^aB^	51.60^aA^	44.38^a^
Tyrosine	O	18.75^aAB^	14.23^aB^	12.58^bB^	25.99^aA^	17.89^a^
	P		18.85^aB^	21.07^aAB^	27.22^aA^	21.47^a^
TEAAs	O	383.36^aB^	400.71^aAB^	440.88^aA^	417.69^aAB^	410.66^a^
	P		411.32^aA^	402.10^bA^	401.23^aA^	399.50^a^
TNEAAs	O	565.74^aA^	550.40^aAB^	509.10^bB^	532.79^aAB^	539.51^a^
	P		537.68^aA^	548.89^aA^	549.39^aA^	550.43^a^
TEAAs/TNEAAs	O	0.68^aB^	0.73^aAB^	0.87^aA^	0.78^aAB^	0.77^a^
	P		0.76^aA^	0.73^bA^	0.73^aA^	0.73^a^

^*^Presented on 100 wet weight; CF, Control formula, ^a, b, c^No significant difference (*p* > 0.05) between any two means within the same column with the same superscripted letters, and ^A, B, C^No significant difference (*p* > 0.05) between any two means within the same row with the same superscripted letters.

These differences suggest that fiber source and inclusion level influenced amino acid balance to some extent, although the overall protein profile remained favorable across all formulations (59). The preservation of amino acid quality after fiber enrichment is nutritionally important because it indicates that the reduction in total protein content did not compromise protein balance (60). In this respect, the LCDBSs may be considered nutritionally adequate snack products, particularly when their enhanced fiber density and improved micronutrient composition are considered together (61).

### Fatty acids profiling of formulated LCDBSs

The fatty acid profiles of LCDBSs were characterized by a predominance of unsaturated fatty acids, with only minor differences among treatments (Table 9). Oleic acid was the major fatty acid in all formulations, ranging from 35.00 to 36.56%, followed by linoleic acid (22.06%−24.90%) and palmitic acid (17.81%−19.57%). Lauric, myristic, and stearic acids were also present in moderate amounts, while short-chain and very-long-chain fatty acids occurred only in trace levels. The summed classes showed that monounsaturated fatty acids (MUFA) ranged from 36.82 to 37.65%, polyunsaturated fatty acids (PUFA) from 23.89 to 26.36%, and saturated fatty acids (SFA) from 36.24 to 39.09%.

**Table 9:** Fatty acids profiling of formulated LCDBSs.

Polyphenolics	Fiber type	Fiber %				Mean
		CF	5%	7.5%	10%	
Butyric acid	O	0.39^aA^	0.41^aA^	0.35^bB^	0.39^aA^	0.39a
	P		0.41^aA^	0.38^aA^	0.39^aA^	0.39a
Caproic acid	O	0.23^aA^	0.19^aA^	0.22^aA^	0.26^aA^	0.23a
	P		0.25^aA^	0.22^aA^	0.21^aA^	0.23a
Caprylic acid	O	0.67^aA^	0.61^aA^	0.66^aA^	0.73^aA^	0.67a
	P		0.72^aA^	0.67^aA^	0.58^aA^	0.66a
Capric acid	O	0.88^aA^	0.77^aA^	0.83^aA^	0.91^aA^	0.85a
	P		0.95^aA^	0.86^aA^	0.81^aA^	0.88a
Lauric acid	O	5.45^aA^	5.11^aA^	5.20^aA^	5.49^aA^	5.31a
	P		5.68^aA^	5.53^aA^	5.07^aA^	5.43a
Myristic acid	O	4.67^aA^	4.57^aA^	4.79^aA^	4.99^aA^	4.76a
	P		5.02^aA^	4.95^aA^	4.61^aA^	4.81a
Pentadecanoic acid	O	0.26^aA^	0.26^aA^	0.28^aA^	0.31^aA^	0.28a
	P		0.29^aA^	0.27^aA^	0.27^aA^	0.27a
Palmitic acid	O	17.81^aA^	18.32^aAB^	19.15^aAB^	19.57^aA^	18.71a
	P		19.16^aA^	19.13^aA^	18.63^aA^	18.68a
Palmitoleic acid	O	0.57^aA^	0.43^aA^	0.47^aA^	0.69^aA^	0.54a
	P		0.53^aA^	0.42^aA^	0.47^bA^	0.50a
Margaric acid	O	0.18^aA^	0.18^aA^	0.20^aA^	0.18^aA^	0.19a
	P		0.23^aA^	0.17^aA^	0.22^aA^	0.20a
Stearic acid	O	4.45^aB^	4.88^bB^	5.06^aA^	5.07^aA^	4.87a
	P		5.02^aAB^	5.08^aA^	5.03^aA^	4.90a
Oleic acid	O	36.56^aA^	35.92^aA^	36.15^aA^	35.83^aA^	36.12a
	P		35.78^aBC^	36.07^aAB^	35.00^bC^	35.85a
Linoleic acid	O	24.10^aA^	24.45^aA^	22.96^aA^	22.06^aA^	23.39a
	P		22.55^aA^	22.72^aA^	24.90^aA^	23.57a
Linolenic acid	O	2.01^aA^	1.91^aA^	1.79^aA^	1.83^aA^	1.89a
	P		1.73^aA^	1.75^aA^	2.18^aA^	1.92a
Arachidic acid	O	0.41^aA^	0.40^aA^	0.41^aA^	0.39^aA^	0.40a
	P		0.39^aAB^	0.40^aA^	0.37^bB^	0.39a
Cis-11-eicosenoic acid	O	0.52^aA^	0.72^aA^	0.64^aA^	0.51^aA^	0.60a
	P		0.51^bA^	0.54^aA^	0.49^aA^	0.52a
Behenic acid	O	0.46^aA^	0.46^aA^	0.47^aA^	0.44^aA^	0.46a
	P		0.43^bAB^	0.46^aA^	0.42^aB^	0.44a
Lignoceric acid	O	0.38^aA^	0.37^aA^	0.38^aA^	0.36^aA^	0.37a
	P		0.35^aBC^	0.37^aA^	0.34^aC^	0.36a
Saturated fatty acids (SFA)	O	36.24^aA^	38.91^aA^	39.09^aA^	38.00^aA^	38.06^a^
	P		36.53^aA^	36.24^bA^	38.49^aA^	36.88^a^
Monounsaturated fatty acids (MUFA)	O	37.65^aA^	36.82^aB^	37.03^aAB^	37.26^aAB^	37.19^a^
	P		37.07^aA^	37.65^aA^	37.03^aA^	37.35^a^
Polyunsaturated fatty acids (PUFA)	O	26.11^aA^	24.28^aA^	23.89^bA^	24.75^aA^	24.76^a^
	P		26.36^aA^	26.11^aA^	24.47^aA^	25.76^a^

^*^Presented on 100 wet weight; CF Control formula, ^a, b, c^No significant difference (*p* > 0.05) between any two means within the same column with the same superscripted letters; and ^A, B, C^No significant difference (*p* > 0.05) between any two means within the same row with the same superscripted letters.

Most of these variations were not statistically significant (*p* > 0.05), indicating that fiber fortification did not materially alter the lipid quality of the snacks (50, 62). This stability is consistent with the fact that the main lipid-contributing ingredients remained essentially unchanged across formulations (63). Maintaining an oleic- and linoleic-acid-rich profile is nutritionally favorable and supports the overall quality of the formulated snacks (62, 64). Importantly, the improvements in fiber density, mineral composition, and sugar reduction were not accompanied by deterioration in fatty acid balance, further supporting the functional value of the LCDBSs (38, 62, 65).

### Postprandial glycemic response of selected LCDBSS in healthy and type 2 diabetes participants

**Figure 1:**
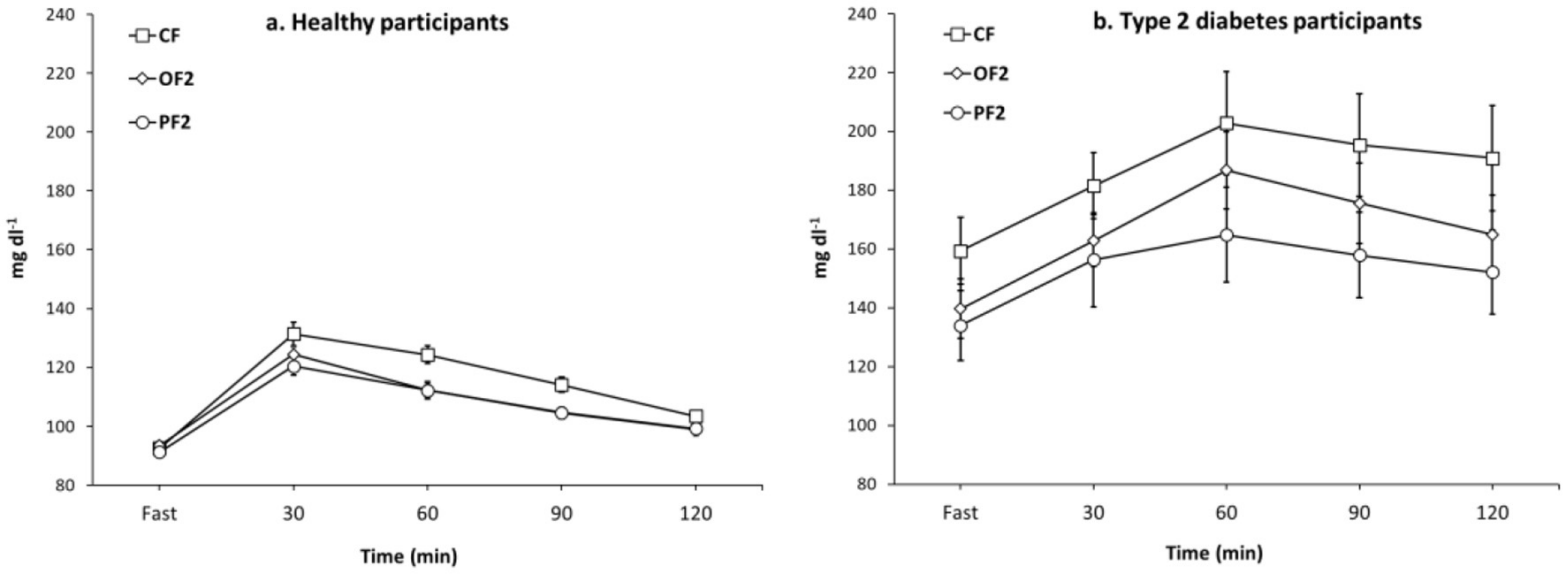
Postprandial glucose response in healthy **(a)** and type 2 diabetes participants, **(b)** after ingestion of 100 g from the control formula (CF), oat bar formula (OF2; 7.5% oat fiber), and psyllium bar formula (PF2; 7.5% psyllium fiber). Glucose concentrations (mg dl1) were measured at fasting and at 30, 60, 90, and 120 min after consumption using the finger-prick protocol; values are presented as mean ± SE.

Data in Figure 1 summarizes the postprandial glycemic responses of selected LCDBSS in Healthy (Figure 1a) and Type 2 Diabetes (Figure 1b) participants. The postprandial glucose responses differed clearly among the three formulas. In both healthy participants and participants with type 2 diabetes, the control formula (CF) produced the highest glucose concentrations, the oat bar formula (OF2) showed an intermediate response, and the psyllium bar formula (PF2) showed the lowest response. In the type 2 diabetes group, glucose peaked at 60 min with CF, whereas OF2 and PF2 produced noticeably smaller rises and lower values at 120 min, indicating a stronger attenuation of the postprandial excursion with psyllium. In healthy participants, the same ranking was observed, although the absolute changes were smaller, reflecting better baseline glycemic regulation.

These findings suggest that both fiber-enriched bars improved postprandial glucose handling, but the psyllium formulation was more effective than the oat formulation (53, 66). This is consistent with psyllium's higher viscosity and stronger gel-forming properties, which can slow gastric emptying, delay carbohydrate digestion, and reduce glucose absorption in the intestine (13, 67). Oat fiber, particularly beta-glucan, also supports glycemic control, but its effect is often more modest and depends on factors such as dose, viscosity, and the food matrix (66). The greater reduction in glucose response with PF2, therefore, fits well with the broader literature showing that psyllium tends to exert a stronger postprandial glycemic-lowering effect than oat fiber (68).

## Strengths and limitations

This study develops low-calorie, fiber-enriched date snacks and rigorously characterizes their proximate composition, minerals, sugars, phytochemicals, amino acids, and fatty acids using standard analytical methods. It then evaluates acute postprandial glycemic responses in both healthy adults and individuals with type 2 diabetes, under controlled, ethically approved conditions, showing that 7.5% psyllium bars most effectively attenuate glucose excursions while maintaining acceptable sensory properties. While key limitations include the small, single-center samples (*n* = 20 per group) and the acute design, which limit generalizability and preclude inferences about long-term glycemic control, body weight, or cardiometabolic risk. Clinical testing was restricted to the 7.5% fiber level, leaving the glycemic dose–response of 5 and 10% formulations unexplored. The study relies on capillary glucose readings without insulin, satiety, or gastrointestinal tolerance outcomes. It allows heterogeneous antidiabetic regimens among participants with diabetes, all of which may confound glycemic responses and limit mechanistic interpretation.

## Conclusions

In conclusion, the developed low-calorie date-based snacks achieved a balance of sensory acceptability, improved nutritional quality, and favorable glycemic performance, with the 7.5% psyllium formulation showing the strongest attenuation of postprandial glucose in both healthy adults and individuals with type 2 diabetes, while the 7.5% oat formulation also outperformed the control. Importantly, the snacks retained high phenolic content and antioxidant activity, a nutritionally favorable amino-acid profile, and an unsaturated fatty-acid-rich lipid composition, indicating that fiber enrichment enhanced metabolic value without compromising key compositional attributes. These findings support the potential of culturally familiar date-based snacks as more diabetes-friendly alternatives, with psyllium's superior effect likely reflecting its greater viscosity, whereas oat fiber still offers a meaningful glycemic benefit. Future research should examine long-term effects on glycemic control, satiety, lipid metabolism, and body weight in larger, more diverse populations, while also optimizing fiber type, dose, and food-matrix interactions, and assessing the bioaccessibility and bioavailability of the preserved phenolics, amino acids, and fatty acids.

## Data Availability

The raw data supporting the conclusions of this article will be made available by the authors, without undue reservation.
